# A scoping review on muscle cramps and spasms in upper motor neuron disorder–two sides of the same coin?

**DOI:** 10.3389/fneur.2024.1360521

**Published:** 2024-03-01

**Authors:** Eva Rudjord Therkildsen, Pernille Kaster, Jens Bo Nielsen

**Affiliations:** ^1^Department of Neuroscience, University of Copenhagen, Copenhagen, Denmark; ^2^Elsass Foundation, Charlottenlund, Denmark

**Keywords:** muscle cramps, muscle spasms, spasticity, upper motor neuron disorder, movement disorder

## Abstract

**Background:**

Muscle cramps are typically regarded as benign muscle overactivity in healthy individuals, whereas spasms are linked to spasticity resulting from central motor lesions. However, their striking similarities made us hypothesize that cramping is an under-recognized and potentially misidentified aspect of spasticity.

**Methods:**

A systematic search on spasms and cramps in patients with Upper Motor Neuron Disorder (spinal cord injury, cerebral palsy, traumatic brain injury, and stroke) was carried out in Embase/Medline, aiming to describe the definitions, characteristics, and measures of spasms and cramps that are used in the scientific literature.

**Results:**

The search identified 4,202 studies, of which 253 were reviewed: 217 studies documented only muscle spasms, 7 studies reported only cramps, and 29 encompassed both. Most studies (*n* = 216) lacked explicit definitions for either term. One-half omitted any description and when present, the clinical resemblance was significant. Various methods quantified cramp/spasm frequency, with self-reports being the most common approach.

**Conclusion:**

Muscle cramps and spasms probably represent related symptoms with a shared pathophysiological component. When considering future treatment strategies, it is important to recognize that part of the patient’s spasms may be attributed to cramps.

## New and noteworthy

A systematic review of the literature underscores the challenges associated with distinguishing between muscle spasms and cramps in upper motor neuron disorder (UMND) due to current measurement methodologies and a limited understanding of their underlying pathophysiology. Clinicians should exercise caution, considering the possibility that specific spastic symptoms noted in neurological patients may be attributable to muscle cramps. This recognition prompts a reconsideration of current treatment strategies.

## Introduction

Muscle cramps and spasms – two words that resonate with most people – yet their distinguishing boundaries are unclear. Muscle cramps in healthy individuals are commonly characterized as sudden, involuntary muscle contractions, accompanied by visible or palpable knotting of muscle, self-limiting within minutes, and relieved by stretching (1). Muscle spasms refer to involuntary muscle contractions and may encompass muscle cramps (2), but in a different setting denote a specific symptom associated with spasticity (3).

Muscle cramps are common in the general population, with a reported incidence ranging from 36 to 95% (4–6), and they are even more pronounced in particular subpopulations (e.g., lower motor neuron disease (7), neuropathies (8), metabolic disorders, pregnancy, elderly people (4) and during strenuous physical exercise (9, 10)). Likely, the etiology is multifactorial and depends on the subgroup in question (8, 9). Though many factors (e.g., older age, increased exercise load, pain, dehydration, electrolyte imbalance, and eventually muscle fatigue) are suggested to increase the susceptibility, cramps ultimately appear to have a neurogenic origin (11, 12). Therefore, it is all the more surprising that muscle cramps are rarely reported in patients with upper motor neuron disorder (UMND) (1) when considering that this population group is expected to be at high risk of muscle cramps due to an altered neuromuscular function and multiple risk factors, i.e., older age, perceived fatigue, and strenuous exercise after periods of inactivity (9, 13). Instead, involuntary muscle contractions. muscle spasms. are frequently reported to impact the function and the daily life of patients with UMND (14). Importantly, muscle spasms are treated with CNS depressing medication (15, 16), whereas cramps are foremost approached with non-pharmaceutical therapies that are unexplored in individuals with neurological disease (17, 18). Thus, considering that current treatment approaches differ, it becomes imperative to investigate the potential underreporting of cramps in UMND.

We raise three explanations: (1) muscle cramps occur less frequently in UMND than in the rest of the population, (2) muscle cramps occur in UMND but are an unobserved, negligible symptom, or (3) muscle cramps are present in UMND, but are described using different terminology, e.g., *muscle spasms* (Supplementary Figure S1). In the following, we argue that muscle cramps and spasms may be conflated and represent partially overlapping phenomena, and if so, this could have significant therapeutic implications.

## Methods

**Figure 1:**
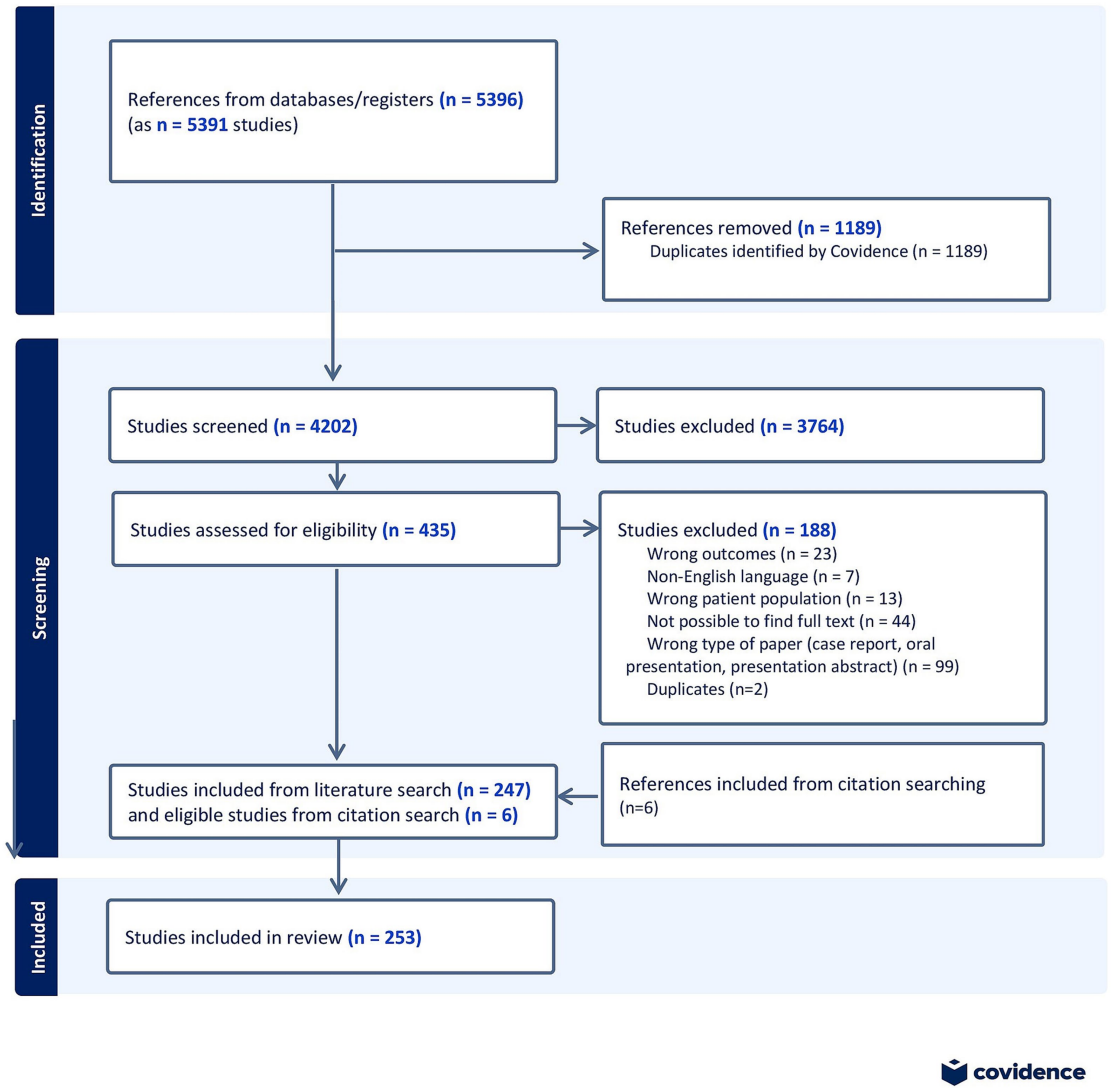
PRISMA flow diagram, showing the process of identification, screening, assessment for eligibility and inclusion of studies in the scoping review.

We used the PRISMA-ScR (PRISMA extension for Scoping Reviews (19) as a framework for this scoping review and its accompanying protocol is provided by the communicating author on request. Figure 1 illustrates the PRISMA flow diagram.

This review intends to answer the following questions, based on the existing scientific literature:

- How frequently do scientific studies investigate muscle spasms and cramps in patients with UMND?
- How are muscle spasms and cramps defined, classified in relation to spasticity, characterized, and measured?

The responses to these questions create a foundation that contributes to addressing our central question: Is it possible that muscle cramps and spasms may be interchanged individuals with UMND–at least in the scientific literature?

### Eligibility criteria

Studies were considered relevant for the review if they (1) addressed either muscle cramps or skeletal muscle spasms and (2) specifically reported cramps and/or spasms in patients with upper motor neuron disease, and (3) were published, full-text available. Studies were excluded if they referred to muscle spasms of the internal organs or facial muscles, reported cramps/spasms as an adverse effect to other treatment, referred to patients with concurrent non-neurological pathology, or were non-English language. Case reports, abstracts, and poster presentations were excluded, whereas all other study types (cohort, cross-sectional, retrospective, RCT, qualitative, review) were included in the scoping review.

### Search strategy

MEDLINE and EMBASE databases were searched in April 2022 for relevant studies, and the search strategy and final search string were reviewed and consulted with an experienced librarian. Suspecting that “muscle cramps” and “spasms” are used interchangeably, we conducted a specific search for these keywords combining MESH-terms, abbreviations, and alternative wording:

((“Spasm”[Mesh]) OR (“Muscle Cramp”[Mesh]) OR muscle cramp* OR leg cramp* OR muscle spasm* OR “exercise associated muscle cramp*” OR EAMC*)

*AND*

((spinal cord injury) OR “multiple sclerosis” OR UMN OR (upper motor neuron*) OR (traumatic brain injury) OR (cerebral palsy) OR (cerebral stroke*) OR (“Motor Neuron Disease”[Mesh]) OR (“Cerebral Palsy”[Mesh]) OR (“Stroke”[Mesh]) OR (“Spinal Cord Injuries”[Mesh]) OR (“Brain Injuries, Traumatic”[Mesh]))

The patient population was limited to individuals diagnosed with damage to the upper motor neurons due to either stroke, traumatic brain damage, multiple sclerosis, cerebral palsy, spinal cord injury. The appearance of both UMND and muscle cramps/spasms in the same citation was ensured using the Boolean operator “AND.” Both search strings for MEDLINE and EMBASE are attached in the Appendix.

### Data extraction and reporting of results

All results (*n* = 5,391) were exported to Covidence. After removing duplicates (*n* = 1,189), the remaining results (*n* = 4,202) underwent abstract screening. This screening process was performed by two independent assessors with expertise in the field. In case of disagreement during the abstract screening, the full text of the study in question was read, and consensus was reached through discussion. Subsequently, all included studies (*n* = 435) underwent full-text review by one of the assessors. Data were extracted from studies that met the eligibility criteria (*n* = 247). Additionally, six additional relevant articles were identified and added to the scoping review through reference list exploration, giving a total of 253 included studies.

In Covidence, a data charting form was utilized, which was developed based on the protocol. The form was refined through an iterative pilot extraction process and thoroughly discussed by both assessors to ensure collection of reliable data. Cases of doubt were reviewed together to reach a common understanding of how to extract data. Extracted information from the studies included main characteristics (title, publication year, study design, patient group), study conclusion, and specific data on spasms/cramps (e.g., use of the term, relation to ‘spasticity’, definition, clinical characteristics, measurement methods, patient’s perceptions, relation to muscle pain, EMG characteristics, and triggering techniques).

Reckoning that ‘muscle cramps’ rarely are described in the literature on UMND, we chose, prior to the extraction process, 12 well-known features (involuntary, painful, measurable by EMG, visible, localized to specific muscles, sudden onset, duration of seconds to minutes, relief through stretching or voluntary antagonist activation, and correlation with exercise, fatigue, sleep, or a shortened state of the muscle) that are associated with cramping in healthy individuals in highly-cited reviews (1, 20). In the following, we refer to these as ‘preselected cramp features’ and use these as a basis for comparison with the descriptions of spasms in the literature on UMND. In addition, the categories “No description” and “Others” were added to the extraction template, and after the extraction process, all “Other” descriptions were analyzed and subgrouped if occurring more than once. All data were exported to Excel (.csv format) for data processing (i.e., grouping and summarizing in tables). For most of the extraction process, there was no room for interpretation, but occasionally, it was difficult to assess the relationship between spasms/cramps and spasticity. In these cases, the study was categorized as ‘others’, and we subsequently evaluated whether new subcategories could be defined. Also, errors were corrected before summarizing data, primarily due to misspellings, mistakes, or inaccurate categorization.

## Results

**Figure 2:**
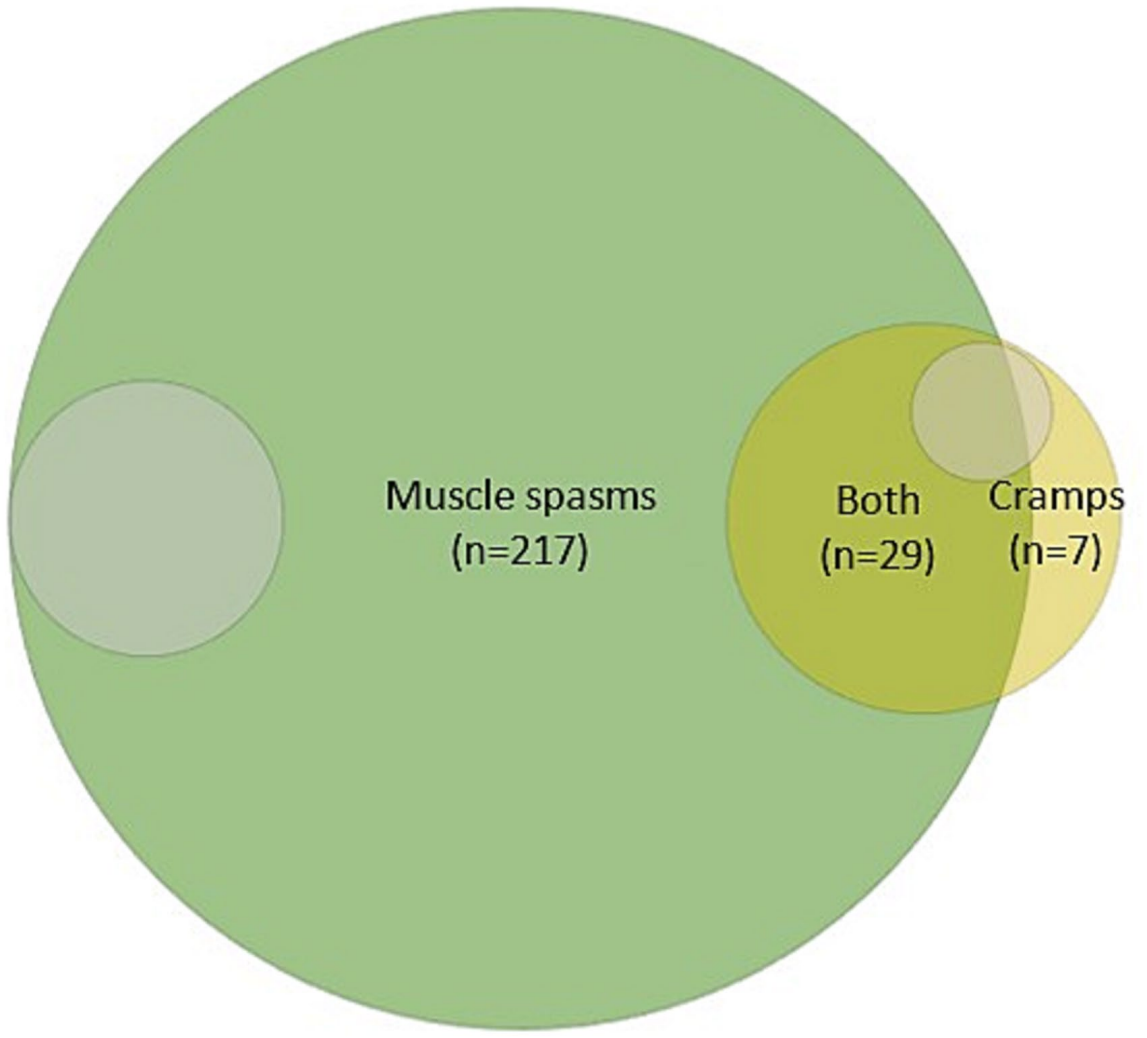
Illustration of the distribution of studies mentioning either spasms, cramps, or both. Grey-shaded areas (~9%) indicate the number of studies in which spasms (*n* = 22) or cramps (*n* = 5) were clearly defined.

The systemic search in Embase and Medline provided 5,391 studies, of which 435 were full-text reviewed, ultimately yielding 253 relevant studies that were included. We found that most studies (*n* = 217) used the term spasm; 7 studies used the term cramp, whereas 29 studies used both terms (Figure 2). Approximately 10 % (*n* = 24) of all studies explicitly defined either the term “spasm” or “cramp.” Of the 29 studies employing both terms, only two studies made a clear distinction between the terms (Summarized in Supplementary Table S2).

The relationship between spasms/cramps and concept of spasticity was interpreted with variability across the scientific literature; however, most studies (*n* = 188) established a connection between the two terms (Supplementary Table S1). Fifty-seven (*n* = 57) studies explicitly delineated ‘spasms/cramps’ from ‘spasticity’ as distinct symptoms, and 10 (*n* = 10) studies addressed the necessity of clearly defining spasticity and its relation to spasms.

### Characteristics

**Figure 3:**
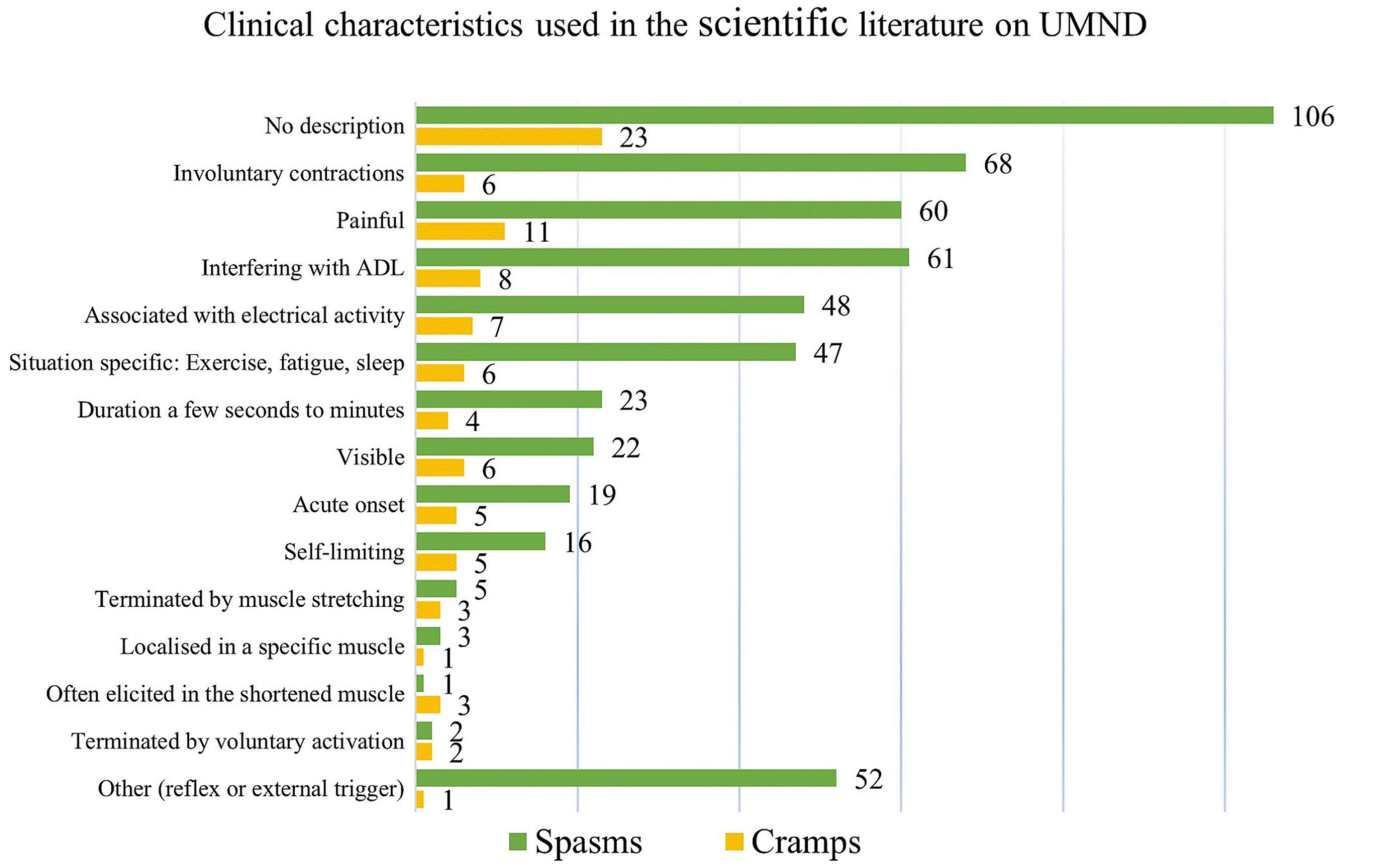
Clinical characteristics that are used to describe either muscle spasms or cramps. Well-recognized features, known from studies in healthy, exercising individuals, were selected prior to the review process. During the subsequent extraction process, we specifically addressed whether similar characteristics were used for muscle spasms. In addition, the categories “No description” and “Others” were added to the extraction template. For spasms. other” comprised the classification of spasms as reflexes, the subdivision of spasms into clonus, flexor, and extensor spasms, and the triggering by specific stimuli (electrical, cold, touch, or passive movement).

Half of the 253 studies (*n* = 129) included for full-text reading had no description of cramps nor spasms. The remaining half provided only sparse descriptions, of which most could be assigned to one of the predefined cramp feature categories (Figure 3). However, as several studies on cramps (*n* = 8) and spasms (*n* = 61) highlighted that the contractions could interfere with everyday activities, this subcategory was added to the bar chart (Figure 3). The category “others” covered the remaining descriptions (*n* = 62) that did not fall within the predefined groupings. These mainly included the classification of spasms as reflexes (*n* = 23) with a subdivision of spasms into clonus, flexor, and extensor spasms, and the triggering by various stimuli (*n* = 28; electrical, cold, touch, or passive movement).

Special attention was given to the relation between the involuntary muscle contractions and pain: We found that 76 (out of 216) studies referred to spasms as ‘painful’, and equivalently 4 (out of 7) studies on cramps. Very often, spasms (*n* = 47) and cramps (*n* = 6) occurred more frequently in a specific setting, such as during sleep, exercise, or fatigue. Supplementary Table S3 (Appendix) provides an overview of the different ways in which cramps/spasms qualitatively are described in relation to exercise, and fatigue.

### Measures of spasms/cramps

**Figure 4:**
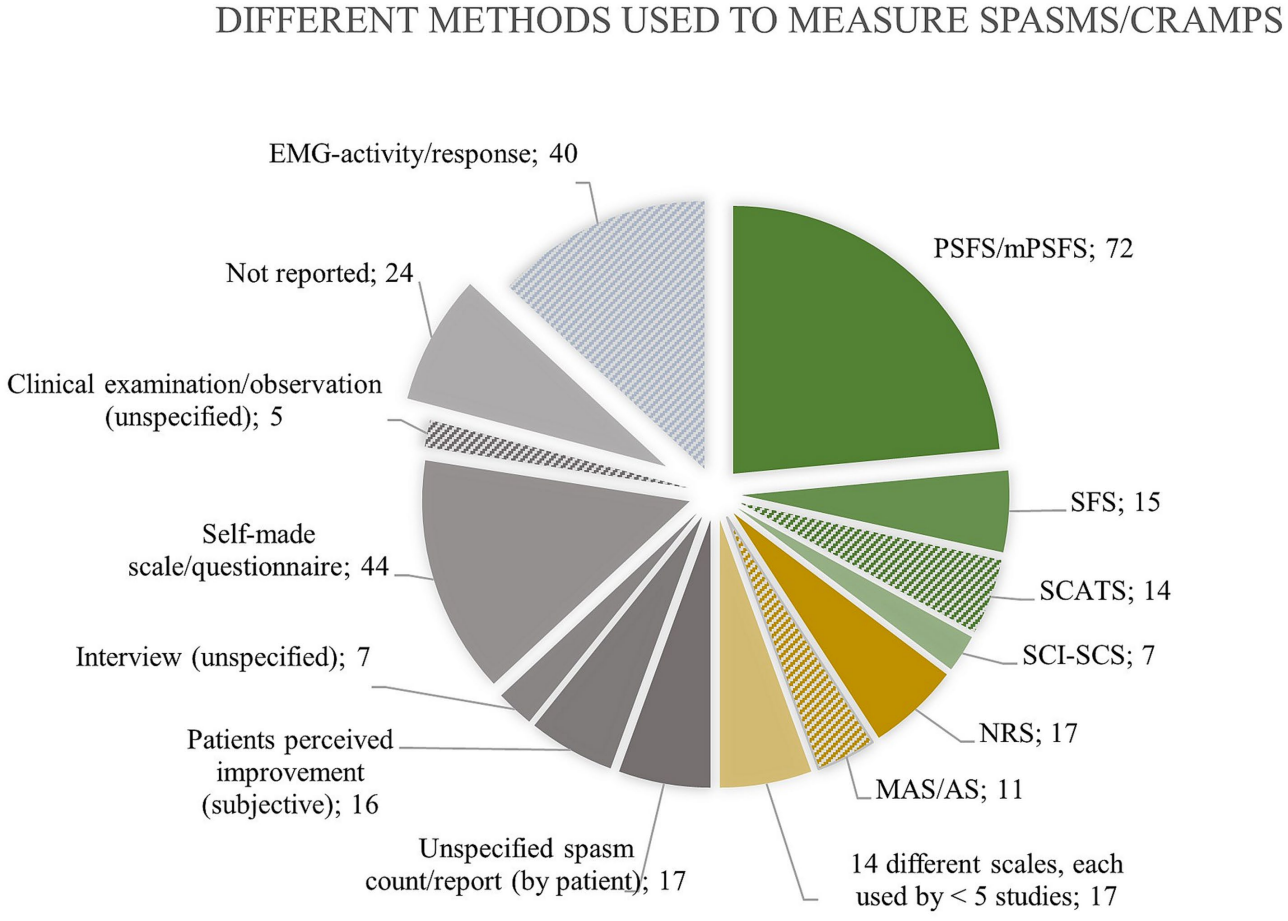
Overview of the different methods used to measure muscle spasms/cramps with indication of the absolute number of studies using each method. Only 108 times (green color), a measurement tool designated for evaluating spasms was used. Forty-five times (*n* = 45, yellow) a tool, not invented for measuring spasms, was used. Thirty-eight times (*n* = 38, blue) electromyography confirmed spasms. The remaining (*n* = 113, grey) either did not report or used a very unspecific measure. Solid color fill indicates that the measure relies on patient perception of “spasms. as opposed to hatched color fill indicating measuring by another examiner. Abbreviations: Penn Spasms Frequency Scale (PSFS), Spasm Frequency Score (SFS), Spinal Cord Assessment Tool for Spastic Reflexes (SCATS), Spinal cord injury Secondary Conditions Scale (SCI-SCS), Numerical Rating Scale (NRS), Ashworth Scale (AS), Modified Ashworth Scale (MAS).

Thirty studies used patient questionnaires to evaluate the prevalence of spasms/cramps in UMND, and the self-reported frequency varied significantly among these surveys (15 to 99%). Various methods (>25 different measures) were used to measure spasms/cramps in UMND (Figure 4). Most measures (*n* = 212; 70%) rely on patients’ perception of muscle spasms, e.g., through Penn Spasms Frequency Scale (PSFS), Spasm Frequency Score (SFS), Numerical Rating Scale (NRS), spasm count, or a self-made scale etc. In 5 % of these studies, in which self-reporting was used as a measure, did the participants receive brief information on the presentation/meaning of cramps/spasms prior to self-reporting.

A measurement tool designated for evaluating spasms was used in 108 studies, of which the PSFS was the most frequently used (*n* = 72). Forty-five studies (*n* = 45) applied a tool, such as Ashworth Scale (AS), H-reflex size, or NRS, that was nor invented for measuring spasms, and 38 studies (*n* = 38) applied electromyography to measure spasms. The remaining studies (*n* = 113) either did not report anything regarding how they measured spasms (*n* = 24) or used a very unspecific measure, e.g., undefined spasm count, scale, or subjective improvement. Figure 4 provides an overview of the numerous methods that are used to quantify spasms/cramps in the literature on UMND.

In 32 of the included studies, spasms/cramps were deliberately triggered, primarily electrically/magnetically (*n* = 17), or via triggers, such as warm, cold, tickling (*n* = 17), or stretch (*n* = 6). Electromyography was, in addition to verifying the presence of spasms, used to determine the stretch reflex (*n* = 16), H/M-ratio (*n* = 15), H-reflex (*n* = 13), cutaneomuscular reflex (*n* = 10), clonus (*n* = 8) etc., and in several studies spasms were correlated to the size of these muscular reflexes.

## Discussion

### In search of an under-recognized symptom

We conducted a systematic literature search, which aimed to explore the definition, prevalence characterization and measurement of cramps and spasms in the literature on UMND. Based on these findings, we evaluated if current clinical and laboratory measures can distinguish muscle cramps from spasms while considering shared pathophysiological mechanisms that could be inferred from the studies.

To investigate an under-recognized phenomenon poses a puzzle. Thus, reckoning that ‘muscle cramps’ rarely are described in the literature on UMND, we used 12 pre-defined, widely-recognized features associated with cramping in healthy individuals as a benchmark for comparing the descriptions of spasms in the literature on UMND.

*In Upper Motor Neuron Disease, muscle cramps “disappear”, and spasms develop*.

The systematic search confirmed our initial hypothesis that ‘cramping’ is confined to specific patient groups other than UMND (1, 2, 21, 22), as illustrated in Figure 2. In addition, less than 10 % of the studies defined either cramps or spasms, and among those covering both terms (*n* = 29 studies), only two differentiated between them. Thus, the literature displays a lack of consensus regarding the semantics, and definition of these.

Critics will argue that this demonstrates a mere case of semantic nitpicking. However, we argue that unclear definitions along with the recognition that only half of the studies describe the muscle contraction in question (Figure 3) contribute to confounding and misleading use of terms.

*Can muscle cramps be distinguished from muscle spasms based on clinical characteristics in the literature?*

*We find that currently, there is no reliable way for clinicians to differentiate between muscle cramps and spasms based on available clinical data in the scientific literature. They share many clinical similarities, including onset, duration, electromyography, susceptibility factors (nighttime, fatigue, and exercise), pain and response to stretching according to patients’ reports.*

Half of all studies (*n* = 129, 51%) lacked explicit descriptions of cramps or spasms, and the remaining studies, while predominantly using the term ‘spasms’, predominately aligned with preselected cramp features. This underscores the significant convergence in characterizing cramps and spasms (Figure 3), reaffirming their clinical resemblance.

Spasms typically persist for a few seconds to minutes, occur spontaneously as verified by EMG (23, 24), or in relation to specific triggers (e.g., touch, movement). Although we find no records on whether patients can voluntarily terminate their spasms (e.g., using stretch or activation of the antagonist), many patients report subjective improvement in spasms (25)/spasticity (26) when doing stretch. Also, spasms often develop under similar circumstances as cramps, such as during the night ‘similar to nocturnal leg cramps’ (27, 28), when subjects are fatigued, or in relation to exercise (Supplementary Table S3).

Notably, the self-reported spasm frequency varies considerably across surveys, potentially due to differing patient interpretations of ‘cramps’ and ‘spasms’, and lack of objective measures. Reviews on cramps propose a differentiation criterion based on the presence of pain in cramps (1, 2, 22). In contrast, a cross-sectional study on 61 patients with spasticity challenges this notion by remarking that *the general concept of “painful” spasms is inaccurate since about two-thirds of the involuntary movements were painless* (29). We find that every third study (*n* = 76) describe spasms as painful, underscoring a possible disagreement across the literature. This discrepancy introduces ambiguity in both scientific literature and patient accounts - and suggests the potential existence of a partially distinct, yet co-occurring, phenomenon such as cramps.

As the association to exercise is a core feature in muscle cramping in healthy individuals (30), we specifically addressed exercise and fatigue in relation to spasms in UMND (Supplementary Table S3).

Following a neurological insult, individuals tend to become less physically active, potentially leading to more fatigable muscles during everyday activities and physiological testing (31).

This perspective aligns with some patients’ perception that *too much activity is associated with fatigue and a further increase in spasticity* (25) and that spasms are linked with *physical overexertion* (29), *extreme fatigue* (32), *uncustomary exertional efforts* (33), *fatigue, overuse, and inflammation* (34). Similarly, cramps in healthy individuals are *associated with exercise, especially with beginning of a new exercise program* (22) or *particularly after periods of inactivity* (13).

The escalation of sensory inputs induced by physical exercise likely plays a role in this correlation, yet specific exercise interventions have demonstrated an immediate exacerbation of spasms with heightened physical exertion (32, 33). Does this imply that individuals with upper motor neuron disease (UMND) should abstain from physical exercise to mitigate the risk of spasms?

In contrast, we posit that individuals with UMND will adapt over time to a consistent training regimen, experiencing reduced muscle fatigue and fewer spasms during daily activities. Addressing this potential impediment is crucial for clinicians recommending exercise to UMND patients. The prospect of a positive, long-term impact of FES-assisted exercise on muscle spasms is suggested by the experiences of individuals with spinal cord injuries (35). Nonetheless, dedicated interventions examining the effect of training on muscle spasms are warranted (33, 34).

*Why is it problematic that the terms ‘spasms’ and ‘cramps’ are used interchangeably?*

*More than twenty-five different methods are used to measure spasms or cramps in UMND, but most rely on self-reported observations, and no measure distinguishes between the cramps and spasms. Thus, misinterpretation may occur due to a lack of reporting guidance, differing perceptions of spasms, cramps, and spasticity between clinicians and patients, and the absence of clinically applicable, objective measures.*

Confusing muscle cramps with spasms may ultimately lead to different treatments, as muscle spasms are often considered part of *spasticity* that arises following CNS damage. Many clinicians use a broad definition of spasticity (36) which places greater demands on specifying the individual spasticity-symptoms, as hypertonia, clonus, hyperreflexia, and spasms can exist independently and do not necessarily share common pathophysiology (37).

Unfortunately, we find that a clear distinction between spasms and spasticity is not made in the studies on UMND, among which only one-fifth (*n* = 57) explicitly demarcate ‘spasms/cramps’ from ‘spasticity’ as distinct symptoms. Research has explored the ambiguity between spasms and spasticity, revealing conflicting expert views on their distinction (38), as well as incongruence between clinicians’ and patients’ vocabulary and understanding of the terms (25).

We speculate that spasms and spasticity, which often go hand in hand, are treated as if it is one and the same. This may explain why clinicians seem more likely to resort to antispastic pharmaceuticals when treating muscle spasms than when treating muscle cramps.

*In brief, conceptual ambiguity is a slippery slope whereby muscle cramps are interpreted as spasms that are interpreted as spasticity.*

The consequence of miscommunication is potentially significant, as the prevalence of cramps and spasms foremost (~50%) are quantified with different variations of self-reported spasm frequency tools (PSFS, SFS, NRS etc.) without aligning interpretations of cramps/spasms before the assessment. We find that there is a need to develop a better way to quantify spasms seeing that one-fifth of the studies do not report how they measure spasms, whereas the remaining use more than 25 different methods, many of these unvalidated (17, 39) and with poor correlation between self-reported spasms and (semi)objective measures (e.g., EMG (23, 40, 41), Spinal Cord Assessment Tool for Spastic Reflexes (SCATS) (14, 42), plantar stimulation response (43), H-reflex depression (44)).

Evidently, similar methodological challenges pertain to the measurement of muscle cramps and spasms, facing that both are challenging to predict and examine. Therefore, spasms/cramps are deliberately elicited through electric/magnetic stimulation or physical manipulations in 32 of the included studies. We highlight those comparable procedures (voluntary activation (45), fatigue (31), nociceptive stimulation, tendon vibration, and electrical stimulation with increasing frequency (46)) are used to provoke both spasms and cramps. Interestingly, the EMG firing response to the external stimulation shows similarities during spasms in UMND (47, 48) and cramps in healthy individuals (49, 50). This suggests a potential mechanistic overlap.

Ultimately, miscommunication and lack of objective measures may lead to false conclusions, as exemplified by studies, which make conclusions on ‘muscle spasms’ but measure another part of *spasticity*, such as increased muscle tone or reflex excitability [e.g., using Modified Ashworth Scale (MAS) or H-reflex size].

Therefore, we stress that experts and patients have very different perceptions of muscle cramps, spasms, spasticity, and their interrelatedness, and we raise the concern that the incidence of misdiagnosis of spasms/cramps in UMND is considerable.

Recognizing that part of the patient’s spasticity may be attributed to muscle cramps necessitates a critical reconsideration of current treatment strategies, seeing that muscle spasms usually are treated with CNS depressing medication (15, 16), whereas cramps primarily are addressed with non-pharmaceutical therapies that are largely unexplored in individuals with neurological disease (17, 18).

Thus, this recognition also emphasizes the future perspective for investigating if part of the involuntary contractions in spasticity may be mitigated using treatment strategies typically employed for muscle cramps. If so, this approach may reduce the reliance on antispastic medication and improve motor rehabilitation efforts that could otherwise be compromised by CNS-depressing medications.

The discussion of this scoping review is constrained by the fact that a search examines how the scientific literature describes muscle cramps and spasms in UMND, and thus it is possible that clinicians in practice have more nuanced understanding of these phenomena. However, it is most likely that the literature and clinical practice reflect each other.

## Conclusion

The systematic literature search reveals a paucity of scientific documentation pertaining to muscle cramps in individuals with upper motor neuron disease (UMND). This observation is significant, especially considering that cramps are notably common among individuals with other neurological impairments and in contexts often experienced by UMND patients. As such, we propose that cramps may indeed be manifest in individuals with UMND but are often misclassified as spasms at least in the scientific literature.

## Author contributions

ET: Conceptualization, Data curation, Formal analysis, Investigation, Methodology, Validation, Visualization, Writing – original draft, Writing – review & editing. PK: Data curation, Investigation, Validation, Writing – original draft, Writing – review & editing. JN: Conceptualization, Supervision, Writing – original draft, Writing – review & editing, Investigation, Methodology.
