# Supplementary material for: A scoping review on muscle cramps and spasms in upper motor neuron disorder–two sides of the same coin?
Source: Front Neurol. 2024 Mar 1;15:1360521. doi: 10.3389/fneur.2024.1360521 (PMC10940373; doi:10.3389/fneur.2024.1360521)
Supplement: Supplementary file 1 [file Data_Sheet_1.pdf]

Appendix

Search strategy MEDLINE:

Date of search: 1.4.2022

(("Spasm"[Mesh]) OR ("Muscle Cramp"[Mesh]) OR muscle cramp OR leg cramp* OR muscle spasm* OR "exercise associated muscle cramp*" OR EAMC*)*
AND
((spinal cord injury) OR "multiple sclerosis" OR UMN OR (upper motor neuron) OR (traumatic brain injury) OR (cerebral palsy) OR (cerebral stroke*) OR ("Motor Neuron Disease"[Mesh]) OR ("Cerebral Palsy"[Mesh]) OR ("Stroke"[Mesh]) OR ("Spinal Cord Injuries"[Mesh]) OR ("Brain Injuries, Traumatic"[Mesh]))*
Results: n = 1512 references

Search strategy EMBASE:

Date of search: 10.4.2022

1.	muscle spasm/
2.	muscle cramp/ or heat cramp/ or leg cramp/
3.	(muscle spasm* or muscle cramp* or leg cramp* or exercise associated muscle cramp* or EAMC).mp. [mp=title, abstract, heading word, drug trade name, original title, device manufacturer, drug manufacturer, device trade name, keyword heading word, floating subheading word, candidate term word]
4.	1 or 2 or 3
5.	cerebral palsy/
6.	cerebrovascular accident/ or ischemic stroke/
7.	traumatic brain injury/ or acquired brain injury/ or brain damage/
8.	multiple sclerosis/
9.	spinal cord injury/
10.	(cerebral palsy or stroke* or traumatic brain injur* or brain damage or multiple sclerosis or spinal cord injur*).mp. [mp=title, abstract, heading word, drug trade name, original title, device manufacturer, drug manufacturer, device trade name, keyword heading word, floating subheading word, candidate term word]
11.	5 or 6 or 7 or 8 or 9 or 10
12.	4 and 11

Results: n = 3122 references

Fig. 1:

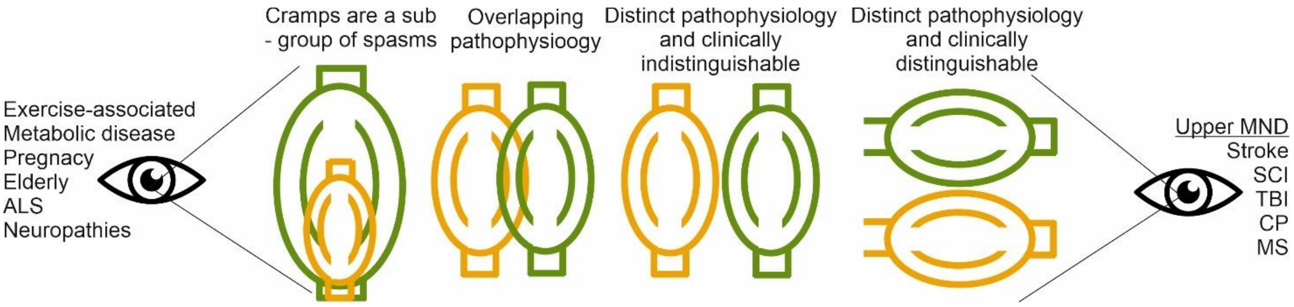

Figure 1: Depending on the clinician's perspective and the setting, involuntary contractions are considered as either muscle cramps (yellow) or muscle spasms (green). We propose the following scenarios that cramps and spasms 1) are partly the same, 2) share similar pathophysiology and clinical presentation 3) have distinct pathophysiology, but same clinical presentation, or 4) have distinct pathophysiology and clinically distinguishable.

Table 1: Relationship between spasticity and spasms/cramps

Relation between spasms/cramps and spasticity	*number*
Spasticity and spasms are used interchangeably	97
Spasms are a manifestation of spasticity or e.g. 'associated with' spasticity)	91
Spasticity and spasms are separate entities	57
Do not mention spasticity in relation to spasms/cramps	22
The study problematises the need for more clear definitions	10

Table 2

Table 2: Summary of all studies, that mentions both 'spasms' and 'cramps' (n=29). Addressing whether each study differentiates between the terms 'spasms' and 'cramps':

	**Relation between muscle spasms and cramps**	**Does the study specify the relation between spasms and cramps?**
Muscular cramp: causes and management (Swash, 2019)	Spasticity is not mentioned. "Muscle spasms" and "cramps" are used as to separate words, but spasms are not defined in relation to cramps.	No
Sleep problems in multiple sclerosis (Tachiana, 1994)	*"Spasticity causing night cramps and flexor spasms."*	No
Health Authorities Data Collection of THC:CBD Oromucosal Spray (Patti, 2016)	*"Spasticity-associated symptoms such as cramps and nocturnal spasms improved in most responding patients"*	No
Sexual problems in multiple sclerosis (Stenager, 1994)	*"Both sexes may experience sexual difficulties due to muscle weakness, paralysis, spasticity, muscle cramps." and "Spasticity, that is rigidity in arms or legs, can cause unpleasant cramps during intercourse"*	No
Chronic intrathecal baclofen administration in severe spasticity (Broseta, 1990)	*"cramping pain"*	No
Comparative Analysis of the Effect of Low-Frequency Repeated Transcranial Magnetic Stimulation and Extracorporeal Shock Wave on Improving the Spasm of Flexor after Stroke (Xu, 2021)	Only mentions spasms and spasticity	No
Theoretical and Therapeutic Implications of the Spasticity-Plus Syndrome Model in Multiple Sclerosis (Bruno, 2021)	*"a typical pattern of muscle tone alteration, known as spasticity, is frequently observed in combination with other signs or symptoms such as spasms, cramps, pain, bladder dysfunction, sleep disturbances, fatigue, and tremor"* and "*spasticity, triggered by central nervous system (CNS) damage, and other symptoms caused by axon demyelination in terms of conduction block (spasticity, fatigue, weakness, and retention) and ephaptic transmission (spasms, pain, allodynia, and urgency) are placed on the same level."*	No
Gabapentin for relief of upper motor neuron symptoms in multiple sclerosis (Mueller, 1997)	*"spasticity and painful muscle cramps were eligible"* and *"spasticity and painful muscle spasms"*.	No
Forces consistent with plateau-like behaviour of spinal neurons evoked in patients with spinal cord injuries (Nickolls, 2004)	"*Some indirect evidence suggests that plateau potential and it is thought that they contribute to muscle cramps."* and "hyper-reflexia was noted as being present if the patient reported regular spasms at least once per week, if spasms were observed in the patients' muscles or recorded on the force or EMG traces..."	No
Role of motoneurons in the generation of muscle spasms after spinal cord injury (Gorassini, 2004)	"excessive and unwanted muscle contractions, i.e. muscle spasms" and "Cramps/nocturnal spasms are a spasticity-related symptom"	No
Beyond fatigue: Assessing variables	The study mentions 'muscle spasms', but investigates	No

associated with sleep problems and use of sleep medications in multiple sclerosis (Bamer, 2010)	'leg cramps': Q: *"leg cramps that interfere with your sleep"*	
Spasticity improvement in patients with relapsing-remitting multiple sclerosis switching from interferon-Î² to glatiramer acetate: the Escala Study (Meca-Lallana, 2012)	*"Spasticity increases from painful cramps, spasms or transient clonus to persistent stiffness as the disease progresses."*	No
Advances in the management of multiple sclerosis spasticity: experiences from recent studies and everyday clinical practice (Pozzilli, 2013)	"*At the patient level, a practical definition of spasticity is as follows: 'An unusual tightening of muscles that feels like leg stiffness, jumping of legs, a repetitive bouncing of the foot, muscle cramping in the legs or arms, legs going out tight and straight or drawing up'*"	No
High false-positive rate of questionnaire-based restless legs syndrome diagnosis in multiple sclerosis (Mery, 2015)	*"cramps and/or muscle spasms"*	No
Warm-needle moxibustion for spasticity after stroke: A systematic review of randomized controlled trials (Yang, 2018)	The study uses the Mesh-terms "spasm*," "cramp,".	No
Sleep Complaints and Sleep Quality in Spinal Cord Injury: A Web-Based Survey (Shafazand, 2019)	Authors mentions spasms, but participants report cramps.	No
Assessment and Measurement of Spasticity in MS: State of the Evidence (Hugos, 2019)	Spasticity manifests in many ways, including spasms, resistance to passive stretch, pain, and perception of tightness.	No
The Value of Real-Time Shear Wave Elastography before and after Rehabilitation of Upper Limb Spasm in Stroke Patients (Liu, 2020)	Muscle cramps generally manifest as increased muscle stiffness, which is positively correlated with active or passive muscle strength.	No
Patterns of Medical Cannabis Use among Patients Diagnosed with Multiple Sclerosis (Guarnaccia, 2021)	spasms and cramps appear to be used interchangeable.	No
Patient Perspectives on the Therapeutic Profile of Botulinum Neurotoxin Type A in Spasticity (Jacinto, 2020)	In the patient questionnaire proposing the following symptoms:*"[] Muscle spasms [] Muscle stiffness/rigidity (including painful cramps*)"	No
Management of intractable spasticity of supraspinal origin by chronic cervical intrathecal infusion of baclofen (Conacalves, 1994)	*"reduction of spasms, amelioration of cramping pain"*	No
A Cross-Sectional Study of the Impact of Spasticity on Daily Activities in Multiple Sclerosis.	The study reports, without specifying: "*The terms spasms and clonus were defined to improve the accuracy of responses*.", but later writes *"Spasms/cramps in the arm/leg"*	No / unclear
Color Doppler ultrasound-guided botulinum toxin type A injection	The study uses mostly "spasms", but later writes that botox can 'relieve cramps'	No

combined with an ankle foot brace for treating lower limb spasticity after a stroke (Ding, 2015)		
Effects of THC/CBD oromucosal spray on spasticity-related symptoms in people with multiple sclerosis: results from a retrospective multicenter study (Patti, 2020)	"*Cramps/nocturnal spasms are a spasticity-related symptom"* and "Other: "spasticity is closely associated either directly or indirectly to a wide array of other symptoms and functional impairments, such as cramps and nocturnal spasms"	No
Integrated Management of Multiple Sclerosis Spasticity and Associated Symptoms Using the Spasticity-Plus Syndrome Concept: Results of a Structured Specialists' Discussion Using the Workmat Methodology (Fernandez, 2021)	"Spasticity and spasms/cramps are also frequent and interconnected symptoms" and *"Correspondence analysis of expert data identified two main MS symptom clusters by joint onset or common pathophysiology: (1) spasticity-spasms/cramps-pain, and (2) ataxia-instability-vertigo"*	No
The Value of Real-Time Shear Wave Elastography before and after Rehabilitation of Upper Limb Spasm in Stroke Patients (Liu, 2021)	"Muscle cramps generally manifest as increased muscle stiffness, which is positively correlated with active or passive muscle strength." "If a spasm cannot be effectively treated for a prolonged period of time, it will continue to increase, resulting in limb muscle atrophy, tendon contracture, and joint deformity."	No
Muscle cramps (Miller, 2005)	"Muscle spasm is a term that refers to any involuntary, abnormal muscular contraction, regardless of whether it is painful." "Muscle cramps are a common problem characterized by a sudden, painful, involuntary contraction of muscle. These true cramps, which originate from peripheral nerves, may be distinguished from other muscle pain or spasm"	Yes
Muscular cramps: Proposals for a new classification (Parisa, 2003)	"*A simple distinction may be made between muscle cramps of neuronal origin and muscle cramps of muscular origin. Therefore, the term ''true'' should indicate a real, involuntary, painful muscle contraction, including all the muscle cramps according to Layzer's definition (1)."*<br>"*In MS, painful muscle spasms (muscle cramps) are the most common paroxysmal attack"*<br>*" For a correct classification, a distinction should be made between the term muscle cramp and other common terms. A muscle spasm is an involuntary, sudden muscle contraction. Thus, it is a generic term that does not necessarily indicate a muscle cramp. For example, a facial spasm cannot be included as a muscle cramp because it is not painful. Thus, a muscle cramp is simply a painful spasm."*	Yes, specifying the difference between cramps and spasm. Also, seems like spasm is an umbrella-term, but also used for specific diseases.
Non-drug therapies for the secondary prevention of lower limb muscle cramps (Hawke, 2021)	"*authors diagnosed commonly used synonyms for cramp (for example, spasm)"* and *"Lower limb muscle cramp was not a measured outcome (spasm in people with spinal cord lesion)"*	Maybe – as if 'spasms' are present in SCI and MS, whereas 'cramps' are present in healthy subjects.

Table 3: Studies that describe the relation between exercise/physical activity/fatigue and muscle spasms/cramps in UMND:

**Included studies that discuss the influence of exercise on muscle spasms/cramps**	**Text passage**
Understanding spasticity from patients' perspectives over time (Bhimani et al., 2012)	*"Some recognized that inactivity leads to tight muscles and stretching helps to relax the muscle. A caveat to this was that too much a activity was associated with fatigue and further increase in spasticity."*
Fatigue in human thenar muscles paralysed by spinal cord injury (Thomas C, 1997)	*"Human thenar muscles paralysed by chronic cervical SCI are more fatigueable than control thenar muscles. They also require higher-than-usual stimulation rates to maintain force output during contractions evoked by supramaximal stimulation at constant or variable rates."*
Perception of lower limb spasticity in patients with spinal cord injury (Fleuren J et al, 2009)	Activities associated with spasticity: 'changing position' (22%), 'transfer' (20.7%), 'daily living' (17.1%), ' being active' (17.1%).
Defining the spectrum of spasticity-associated involuntary movements (Abboud H et al, 2019)	*57.4% were kinesogenic and 52.4% were stimulus-sensitive (temperature, resting, transfer/weight-bearing, tactile, stretching/yawning and physical overexertion)*
The clinical features of spasms in patients with a cervical cord injury (Kawamura J et al, 1989)	More frequent: Just after waking in the morning and during Sunday night. Less frequent: after exercise. Always triggered: yawn, cough, excitement, VA, touch, passive movement.
Feasibility and efficacy of home-based neurofunctional exercise vs. resistance exercise programs for ambulatory disability of multiple sclerosis patients with cognitive impairment (Mardaniyan M et al, 2022)	*During 8 weeks home-based strength training (but not neurofunctional training), there were reports of pain, muscle cramps, and extreme fatigue among HBRT participants.* No description of the cramps, reported by the patients.
Long-term user perceptions of an implanted neuroprosthesis for exercise, standing, and transfers after spinal cord injury (Agarwal et al, 2003)	*"All respondents reported a decreased frequency of spasms in the leg muscles, although this may be accompanied by an increase in spasm strength. Eight participants felt that exercising would help them to stand and walk, six felt that it reduced leg spasms, seven felt that it made their legs look good, and five felt that exercise with the neuroprosthesis generally made them feel good."*
Comparison of the effects of body-weight-supported treadmill training and tilt-table standing on spasticity in individuals with chronic spinal cord injury (Adams M et al, 2011)	*The participants in the present study exerted efforts that were uncustomary for them and, in some cases, an increase in spasm severity during the intervention period was reported.*
The Impact Of Sports Activities On Quality Of Life Of Persons With A Spinal Cord Injury: The Impact Of Sports Activities On Quality Of Life Of Persons With A Spinal Cord Injury (Kljajić D et al, 2016)	*Pertaining to the secondary health conditions, sports activities affect the presence or absence of pain and the subjective feeling of poor circulation.* (There is no direct conclusion on spasms or spasticity).
Neurogenic cramps (Katzberg H, 2015)	*"Muscle cramps can occur in anyone under sufficient physiologic stress. A common stressor is strenuous exercise, particularly after periods of inactivity." "Neurogenic cramps have also been described to occur more frequently in patients with central neurological conditions such as Parkinson's disease, stroke and multiple sclerosis, which may occur due to a variety of mechanisms including spinal disinhibition or mechanical factors such as immobility."*
Classification of pain in children with cerebral palsy (Vinkel M, 2022)	*"Pain itself may lead to an increase in muscle spasms, creating a vicious cycle. Other common causes of increased muscle spasms are fatigue, overuse, and inflammation"*
